# Silver-doped bioactive glass fibres as a potential treatment for wound-associated bacterial biofilms

**DOI:** 10.1016/j.bioflm.2023.100115

**Published:** 2023-05-10

**Authors:** Sandeep Shirgill, Gowsihan Poologasundarampillai, Sara Jabbari, John Ward, Sarah A. Kuehne

**Affiliations:** a University of Birmingham, School of Dentistry, 5 Mill Pool Way, Birmingham, West Midlands, B5 7EG, United Kingdom; b University of Birmingham, School of Mathematics, Birmingham, West Midlands, B15 2TT, United Kingdom; c Loughborough University, Department of Mathematical Sciences, Epinal Way, Loughborough, Leicestershire, LE11 3TU, United Kingdom

**Keywords:** Bioactive glass, Antibacterial activity, Silver, Chronic wound biofilms, *Pseudomonas aeruginosa*

## Abstract

Chronic wounds are a drain on global health services and remain a major area of unmet clinical need. Chronic wounds are characterised by a stable and stubborn bacterial biofilm which hinders innate immune response and delays or prevents wound healing. Bioactive glass (BG) fibres offer a promising novel treatment for chronic wounds by targeting the wound-associated biofilm. In this study, the antimicrobial properties of silver-doped BG fibres were tested against *Pseudomonas aeruginosa* biofilms, which are commonly found in chronic wound infections. Results showed that BG fibres doped with silver resulted in a 5log10 reduction in biofilm formation whereas silver-free fibres only reduced formation by log10, therefore silver-doped fibres possess stronger antimicrobial effects. Moreover, there appeared to be a synergistic effect between the fibres and the silver as the application of the silver-doped fibres placed directly in contact with the forming biofilm resulted in a higher reduction in biofilm formation compared to treatments either: using the dissolution ions, using BG powder, or when the fibres were placed in an insert above the biofilm, inhibiting physical contact, instead. This suggests that the physical properties of the fibres, as well as silver, influence biofilm formation. Finally, results demonstrated that silver chloride, which is not antimicrobial, forms and the concentrations of antimicrobial silver species, namely silver ions and nanoparticles, reduce over time when fibres are soaked in cell culture media, which partially explains why the silver-doped dissolution ions contained lower antimicrobial activity compared to the fibres. As silver chloride is more likely to form with increased temperature and time, the antimicrobial activity of silver-containing dissolution ions is highly dependent on the length of ageing and storage conditions. Many studies investigate the antimicrobial and cytotoxic properties of biomaterials through their dissolution products. However, instability of antimicrobial silver species due to silver chloride formation and its effect on antimicrobial properties of silver-based biomaterials has not been reported before and could influence past and future dissolution-based assays as results showed that the antimicrobial activity of silver-based dissolution ions can vary greatly depending on post processing steps and can therefore produce misleading data.

## Highlights

• Anti-biofilm effects of silver-doped bioactive glass fibres were tested.
• Anti-biofilm effects of silver-doped bioactive glass depend on the glass structure.
• The formation of silver chloride/sulphide was investigated.
• Chlorination has an effect on anti-biofilm activity.

## 1 Introduction

Chronic wounds, principally pressure sores, venous leg ulcers and diabetic foot ulcers, are a drain on global health services and remain a major area of unmet clinical need [1], where the annual cost of managing these wounds and associated comorbidities in the NHS was estimated at around £5.3 billion in 2015 [2]. While the rapidly expanding diabetic and elderly populations mean that the incidence of chronic wounds is set to rise, treatment options remain limited and ineffective.

Moreover, multi-species biofilms, which consist of densely aggregated colonies of bacteria encased by a matrix of extracellular polymeric substances (EPS), are abundant in chronic wounds, where studies have found that over 60% of chronic wound samples contained biofilms [3]. This is due to ideal biofilm growth conditions, such as eukaryotic cell death and tissue necrosis caused by tissue hypoxia or anoxia [4], which bacteria can then consume. Biofilm infections induce chronic inflammation and impair re-epithelisation and granulation tissue production [5,6], therefore further delaying/inhibiting successful wound healing. Hence, there has been an increased focus on biofilm-based wound care, where a chronic wound is treated by first targeting the biofilm infection present, for example, using debridement, systemic antibiotics, topical agents or bacteriophages [5]. Previous research showed that using biofilm-based wound care led to complete healing for 77% of chronic wounds in a limb with critical limb ischemia, deducing that “effectively managing the biofilm in chronic wounds was an important component of consistently transforming non-healing wounds into healable wounds” [7].

Unfortunately, biofilms are more tolerant to antimicrobial treatment than their planktonic counterparts due to.

1. Difficulty of antimicrobial diffusion throughout the biofilm;
2. Physiological heterogeneity in the bacterial population due to substrate limitation in the biofilm;
3. The presence of a sub-population of persister cells in the biofilm that evade antimicrobial effects;
4. Bacteria employing an adaptive response, e.g. the use of efflux pumps or the production of antibiotic-degrading enzymes [8].

Consequently, treatments tackling biofilm infections are often unsuccessful leading to the persistence of chronic infections.

Bioactive glass (BG), which is conventionally used for bone regeneration [9,10], could provide a promising treatment for chronic wounds. Their high bioactivity derives from the large amounts of calcium present, which results in an apatite formation when BGs are placed in body fluid due to the deposition of calcium phosphate on the glass surface [11]. More recently, the wound healing properties of BG have been investigated, where it was reported that BG can help stimulate angiogenesis, fibroblast proliferation and granulation tissue production [9,[12], [13], [14], [15], [16], [17], [18]]. Furthermore, BGs with a fibrous structure can enhance wound healing as they have a larger surface area to volume ratio compared to traditional BG scaffolds, resulting in faster dissolution rates when immersed in fluid; therefore increasing the bioactivity of the BG. Moreover, the inherent porosity of the fibres means that skin cells, such as fibroblasts, immune cells and keratinocytes can infiltrate and migrate through them, helping to promote wound healing [19].

Additionally, they are relatively cheap and easy to apply in a clinical setting as opposed to established treatments such as negative pressure wound therapy or skin scaffold application [20,21], where it has been reported that treatment of chronic wounds using the commercially available BG fibres, Mirragen®, was much less than half the cost of cellular and/or tissue-based products [22].

The focus here will be on their antimicrobial properties, where research has found that dissolution products from BGs demonstrate antimicrobial activity against the bacteria: *Pseudomonas aeruginosa*, *Staphylococcus aureus*, *Acinetobacter baumannii* and the fungi *Candida albicans*, all of which can cause major causes of drug-resistant nosocomial infections [23]. Various BG structures, such as microparticles, monoliths and foams, are well known to have multiple possible antimicrobial mechanisms of action. These structures are known to increase the local pH, increase osmotic pressure, cause physical damage by cutting or piercing the organism's cell wall, or change the local environments ionic composition [[24], [25], [26]].

Additionally, doping bioactive glass with metal ions such as silver (Ag), gallium (Ga) and cerium (Ce) can further enhance their antimicrobial properties [15,16,23,[27], [28], [29], [30], [31], [32], [33], [34]].

Silver-based treatments are becoming increasingly popular due to their efficacy against antibiotic-resistant strains of bacteria [35,36] and their ability to eradicate biofilms more effectively than common antibiotics [37]; therefore they potentially provide a viable treatment for infected chronic wounds.

The antibacterial effects of silver ions have been widely investigated and have been found to penetrate the pepti-doglycan cell wall of bacteria, followed by damage to DNA and bacterial proteins involved in key metabolic processes, resulting in a lack of bacterial replication and, ultimately, cell death [38]. Thanks to their non-specific mechanism of action, it is more difficult for bacteria to develop resistance to silver. Silver is already used in the clinical setting, mainly incorporated into wound dressings [39]. Silver nanoparticles are also commonly used for antimicrobial treatment as they are antimicrobial through the release of Ag^+^ ions [40], membrane disruption, and inhibition of bacterial signal transduction [41].

However, one of the main setbacks of using either silver ions or silver nanoparticles for treatment is their ability to transform via oxidation, photoreduction, sulphidisation and chlorination [42]. The formation of silver sulphide and/or silver chloride reduces silver's antimicrobial efficacy as it reduces the concentration of available Ag^+^ ions. Wound exudate and blood plasma contain large concentrations of salts such as sodium and potassium chloride [43,44]. Moreover, common cell culture media often contain both chlorine and sulphur. Therefore, if the formation of silver chloride/sulphide occurs faster than interactions between Ag^+^ ions and bacteria in a wound, treatment may not be successful.

To our knowledge, this is the first study to investigate the anti-biofilm effects of Ag-doped BG with a fibre structure, where previous studies have researched antimicrobial properties of other types of BG structures [28,30,31], including on bacterial biofilms [33]. From experiments, we found that silver chloride forms when Ag-doped BG fibres are soaked in cell culture media Roswell Park Memorial Institute (RPMI) 1640. Silver chloride formation increases with ageing and temperature of storage conditions, reducing their antimicrobial activity against *P. aeruginosa*. Finally, there appeared to be a synergistic interaction between the fibre structure and the silver released from the fibres on the anti-biofilm activity as Ag-doped fibres placed directly on top of forming biofilm resulted in a higher reduction in biofilm formation compared to treatment with undoped fibres and the dissolution ions from Ag-doped fibres alone.

## 2 Materials & methods

### 2.1 Materials

*Pseudomonas aeruginosa* strain PA01-N was kindly provided by Paul Williams, University of Nottingham. Brain heart infusion (BHI) broth and agar were purchased from Sigma Aldrich. Whatman Nuclepore® polycarbonate membranes (0.1 μm pore size, 19 mm diameter) were purchased from Merck. RPMI 1640 was purchased from Gibco. TEM grids, 25% EM grade glutaraldehyde and UA-zero were purchased from Agar Scientific. Alvetex™ scaffold 12-well inserts were purchased from Reprocell. Silver nitrate (99%+ ACS reagent) was purchased from Acros Organics. Acetone was purchased from Fisher Chemical. Ethanol was purchased from Fisher Scientific. Nitric acid 69% was purchased from Astar. Sodium cacodylate (0.2 M, pH 7.4) was purchased from Bioworld. All other reagents were purchased from Sigma-Aldrich®.

### 2.2 Methods

#### 2.2.1 Sol-gel glass powder/fibre production

To make the sol, 1.7 ml TEOS, 3.7 ml ethanol and 7.2 ml 1 M HNO_3_ were mixed, in that order, to a final volume ratio of 17:37:72. After 1 h of stirring, 3.29 g Ca(NO_3_)_2_.4H_2_O was added to obtain a 25.4% (w/v) solution and stirred for a further hour. The 70S30C (70 mol% SiO_2_, 30 mol% CaO) precursor solution was then left to ageat 37 °C for 24 h.

To produce BG powder, a thin layer of the aged sol was poured into a Petri dish and left to dry. The dried sol was then ground down to form a powder.

To produce BG fibres, the binder solution was obtained by mixing 1 g of Butvar® B-98 per 100 ml ethanol until dissolved. The precursor and binder solutions were then mixed in equal volumes, loaded into a metallic needle (22-gauge) and spun on the Tong Li Tech Electrospinning unit. The fibres were collected on aluminium foil positioned below the capillary. The fibres were subsequently transferred to an oven and heat treated at 300 °C for 1 h and then 650 °C for 5 h.

In order to produce glass formulations doped with 2 mol% silver (named 70S28C2A), the sol-gel reaction was carried out as described above, however, following the addition of 3.07g of Ca(NO_3_)_2_.4H_2_O the solution was stirred for 30 min and then 0.32g AgNO_3_ and 66.8 μl H_2_O (to keep the amount of water constant) was added. The solution was stirred for a further 60 min and then aged at 37 °C for 24 h.

#### 2.2.2 Characterising dissolution products from BG fibres

70S30C and 70S28C2A fibres were placed in RPMI 1640 in the ratio of 1.5 mg/ml and incubated at 37 °C. At specific time points (t = 0 min, 10 min, 30 min, 1 h, 2 h, 8 h, 1 day, 2 days, 3 days & 7 days), 100 μl aliquot of dissolution products were removed from the sample and diluted 1:100 in reverse osmosis (RO) water. The concentration of silicon (Si), calcium (Ca), and silver (Ag) in the dissolution products were measured using the PerkinElmer Optima 8000 model for induced coupled plasma optical emission spectroscopy (ICP-OES).

To test for changes in pH in the RPMI 1640, the pH of the entire dissolution products was measured at each time point using a Fisherbrand accumet AB150 pH meter.

#### 2.2.3 Characterisation of BG fibres

Plain BG fibres (70S30C) and BG fibres doped with 2 mol% silver (70S28C2A) were soaked in RPMI 1640 for 7 days. Fibres were then removed and washed with RO water and acetone to terminate any ongoing reaction. Fibres were then placed in an oven for 24 h at 100 °C to dry out.

Approximately 5 mg of fibres were crushed into a powder, these samples were then used for X-ray diffraction (XRD) using the PANalytical Empyrean. XRD analysis was performed on the software DIFFRAC.EVA (V4.3), where crystal identification was done by using the Crystallography Open Database [45].

For transmission electron microscopy (TEM), 5 mg of the crushed fibres were placed in 1 ml of *>*99% ethanol and sonicated for 1 min to suspend the powder. A drop of the suspension was then placed on a TEM grid (lacy carbon film, 200 mesh Au) and left to evaporate. Imaging was performed using the JEOL JEM-2100, using a beam current of 105–110 μA and a voltage of 200 kV. Images were captured using RADIUS Emesis software (RADIUS 2.0). Additionally, selected area electron diffraction (SAED) patterns were captured using a camera working distance of 30–40 cm. Diffraction patterns created using SAED were processed and d-spacings were calculated using the TEM suite in Fiji ImageJ2 (V2.3.0) (see the macro in appendix A for processing steps). Finally, calculated d-spacings were used to identify potential crystal structures using the Materials Project database [46].

#### 2.2.4 Testing the effect of fibres vs dissolution products on biofilm inhibition

To test the effects of the BG fibres on *P. aeruginosa* biofilm formation, Nuclepore® polycarbonate membranes were inoculated with an overnight culture of PA01-N, which was diluted to OD_600_ = 0.05 and placed in wells of a 12-well plate containing 1.5 ml of BHI agar. Wells containing inoculated membranes were then filled with either: 1 ml of RPMI 1640 (for 24 h biofilms); 1 ml of dissolution products; or 1 ml of RPMI 1640 with 10 mg of fibres. Both 70S30C and 70S28C2A fibre compositions were tested. The well plate was then incubated for 24 h at 37 °C. Membranes were then removed from wells, placed in 2 ml of BHI broth, and vortexed for 1 min to resuspend any biofilm formation. The Miles and Misra method was used on resuspension to determine the CFU/ml of the biofilm [47]. Briefly, 200 μl of suspension was placed in the top row of a 96-well plate and 180 μl of BHI broth was placed in all remaining rows underneath. Then 10-fold dilutions were done by removing 20 μl of suspension from the top row and mixing it into the well just below, containing only BHI. This was repeated for all remaining rows. Finally 20 μl of each dilution were placed on an agar plate in triplicate, which was then incubated overnight at 37 °C. Cells were counted and CFU/ml was calculated based on the cell count and dilution factor. Each condition was performed in triplicate with 3 biological repeats.

#### 2.2.5 Measuring the concentration of silver species in dissolution products over time

To measure the concentration of silver species in the dissolution products used for antimicrobial studies 70S28C2A fibres were soaked in RPMI 1640 (with a ratio of 10 mg/ml) for 3 days and then filter-sterilised using a 0.22 μm filter and incubated at 37 °C. At time points t = 0, 1, 3 & 7 days, 2 ml of the dissolution products were ultracentrifuged, using the Sorvall MTX 150 Micro-ultracentrifuge from Thermo Fisher Scientific, at 35,000 rpm for 30 min. This was to separate the silver ions from silver nanoparticles and silver chloride. The supernatant was mixed with 2 ml of RO water and 2 ml of 32% ammonium hydroxide. This was diluted 1:100 in RO water. To collect the silver nanoparticles, the pellet formed from ultracentrifugation was resuspended in 5 ml of 10% nitric acid and was then centrifuged using the Hettich Zentrifugen Universal 320r at 4000 rpm for 1 h to dissolve the nanoparticles in the supernatant. This was then diluted 1:100 in RO water. The silver ion concentration and nanoparticle concentration were measured using the PerkinElmer Nexion 300X model for induced coupled plasma mass spectroscopy (ICP-ms). Three experimental repeats were performed.

#### 2.2.6 Testing the effect of solution ageing and temperature on antimicrobial activity of BG fibres

A frozen stock of PA01-N was used to grow colonies on BHI agar plates by incubating inoculated plates overnight at 37 °C (Thermo Scientific Heratherm incubator). Overnight cultures were grown by inoculating 5 ml of BHI broth with 3 PA01-N colonies, which were then incubated in a shaking incubator (N-BIotek NB-205) at 37 °C and continuously shaken at 110 rpm for 18–24 h.

The antimicrobial activity of dissolution products was measured after 0, 1, 2, 3 & 7 days of incubation. At each time point and temperature, 180 μl of dissolution product was placed in wells of a 96-well plate (in triplicate), followed by 20 μl of the overnight PA01-N culture diluted to an OD_600_ = 1, using the JENWAY 7315 spectrophotometer (to reach an approximate final OD_600_ = 0.1). These were then placed in a shaking incubator for 24hrs at 37 °C and 100 rpm. The bacterial growth in the wells was measured using the Elisa ELx800 plate reader to determine the OD. Three biological repeats were performed.

To collect dissolution products from BG fibres, 50 mg of either 70S30C or 70S28C2A BG fibres were placed in universal tubes with 5 ml of RPMI 1640 cell culture media to obtain a final concentration of 10 mg/ml. Fibres and media were vortexed for 1 min and then left to incubate at 37 °C 110 rpm for 3 days. Dissolution products were filter-sterilised using a 0.2 μm filter and kept in universal tubes. Dissolution products were then either incubated at 4 °C or 37 °C for 0, 1, 2, 3, or 7 days before testing their antimicrobial properties. Dissolution products were then either incubated at 4 °C or 37 °C.

#### 2.2.7 Biofilm treatment using BG fibres or BG powder

Nuclepore® polycarbonate membranes were inoculated with an overnight culture of PA01-N, which was diluted to OD_600_ = 0.05 and placed in wells of a 12-well plate containing 1.5 ml of BHI agar. Wells containing inoculated membranes were then filled with either: 1 ml of RPMI 1640 as a growth control; 10 mg of BG fibres directly contacting the inoculated membrane; BG fibres in an insert just touching the inoculated membrane; or BG powder of the same composition in direct contact with the biofilm. The well plate was then incubated for 24 h at 37 °C. Membranes were then removed from wells, placed in 2 ml of BHI broth, and vortexed for 1 min to resuspend any biofilm formation. The Miles and Misra method was used on resuspension to determine the CFU/ml of the biofilm [47]. Each condition was performed in triplicate with 3 biological repeats.

### 2.3 Physical effect of BG fibres on bacteria

An overnight culture of PA01-N was grown and diluted down to an OD_600_ = 0.1.70S30C fibres were added to the diluted culture to a final concentration of 10 mg/ml and were then vortexed. The culture and fibre mixture was incubated at 37 °C for 1 h.

For scanning electron microscopy (SEM) imaging, 200 μl was placed on a polycarbonate membrane and incubated at 37 °C for 1 h. To fix the sample, 1 ml of 2.5% EM grade glutaraldehyde in 0.1 M sodium cacodylate buffer (pH 7.3) was added to the sample for 10 min and then removed. The sample was dried by dehydrating in ethanol solutions of increasing concentrations (from 20% to 100%) for 10 min each. Finally, HMDS was applied just to cover the sample and left to airdry overnight. Samples were then imaged using the Zeiss EVO MA10 SEM with a voltage of 15 kV and a working distance of 11 mm.

For TEM imaging, 20 μl was placed on a TEM grid and incubated at 37 °C for 1 h. To fix the sample, 20 μl of 2.5% EM grade glutaraldehyde in 0.1 M sodium cacodylate buffer (pH 7.3) was added to the sample for 1 min and then removed. The sample was left to airdry overnight and stained using a drop of UA-zero. Samples were imaged using the same microscope and settings used in subsection 3.1.3.

### 2.4 Statistical analyses

Statistical significance of data was calculated using 1-way ANOVAs, where b × p-value ≤ 0.05, 0.005, 0.0005 was represented by, *, **, *** respectively. Here b was calculated using the Bonferroni correction to correct for multiple comparisons.

## 3 Results & discussion

### 3.1 Dissolution from fibres

**Fig. 1:**
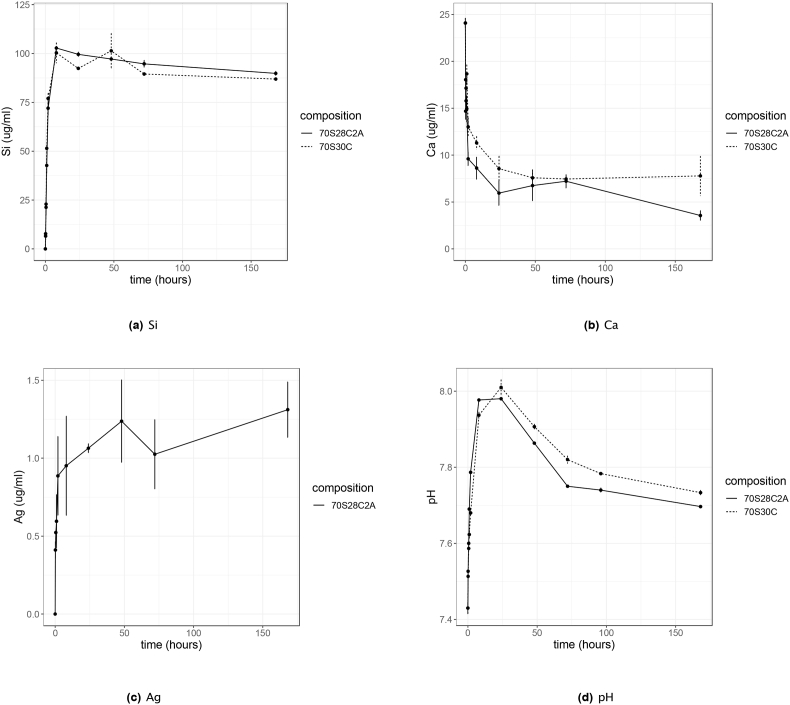
Silicon species (Si), calcium (Ca) and silver (Ag) and changes in environmental pH during fibre dissolution up to 7 days. Both 70S30C and 70S28C2A fibres are soaked in RPMI 1640 in the ratio of 1.5 mg/ml. Error bars represent the average value ± 1 standard error of the mean.

Both undoped fibres and Ag-doped fibres exhibited similar ion release profiles when soaked in RPMI 1640, for both Si and Ca (see Fig. 1). For the release of Si, there was an initial burst release over the first 8 h (Fig. 1a). Ag also demonstrated a burst release, but at much smaller concentrations, over the first few hours, but there was also a slower release of ions thereafter, however, there was a large variation between repeats (Fig. 1c). For both compositions, similar amounts of Si were released but for 70S30C fibres, more Ca remained in the solution. This could be due to the higher concentration in the fibres initially, where 70S30C fibres contain 30mol% of CaO whereas 70S28C2A only contains 28mol% CaO. However this difference in concentration was insignificant according to statistical analysis using a 1-way ANOVA. Both compositions also have similar effects on the pH of the media (Fig. 1d); there was a sharp increase in pH over the first 8 h due to ion exchange between H^+^ ions from the media and Ca^2+^ ions from the fibres. After ion release, precipitation occurred on the fibre surface, therefore these released Ca^2+^ ions, as well as Ca^2+^ ions already in the media were rapidly removed from the environment due to the formation of a hydroxycarbonate apatite (HCA) layer (Fig. 1b), where previous research has found that HCA starts to form on 70S30C BG fibres by 12 h of soaking in simulated body fluid [19]. The pH then slowly declined again. The pH of the 70S30C fibres was slightly higher than the 70S28C2A fibres due to the larger concentration of Ca^2+^ ions in these fibres.

From these results, we can conclude that the addition of silver to the BG fibres has little effect on the release profiles of Si and Ca and on the pH of the environment. The release profile of Ag was used to calculate the approximate concentrations of Ag involved in the characterisation and antimicrobial experiments in the rest of this study.

### 3.2 Fibres vs dissolution products on biofilm inhibition

The antimicrobial effect of dissolution products obtained from both undoped fibres and Ag-doped fibres on inhibiting the growth of PAO1-N bacteria is displayed in Table 1. Both the undoped and doped dissolution products significantly reduced bacterial growth, where p-values for undoped and Ag-doped fibres were 2.87 × 10^−15^ and 8.87 × 10^−8^ respectively. Reduced growth could be due to either the higher pH of the dissolution products compared to the RPMI 1640 used for the growth control or the rise in osmotic pressure. Furthermore, the Ag-doped fibres possessed stronger antimicrobial effects than the undoped fibres. This was due to an additional antimicrobial effect provided by released silver in the dissolution products.

**Table 1:** The average growth of planktonic PAO1-N bacteria after 24 h either untreated or treated with dissolution products from either 70S30C or 70S28C2A fibres. Results show the average OD *±* 1 standard error of the mean. Statistical significance was calculated using a 1-way ANOVA, where b × p-value ≤ 0.05, 0.005, 0.0005 is represented by, *, **, *** respectively (where b = 2 using the Bonferroni correction to correct for multiple comparisons).

Treatment	Growth (OD_600_)
No treatment (24hrs growth)	0.985 *±* 0.01
70S30C	0.497 *±* 0.028
70S28C2A	0.005 *±* 0.003

From Table 1 and it can be observed that the dissolution products of both undoped and Ag-doped fibres can successfully inhibit the growth of PAO1-N bacteria. However, bacteria in this experiment were in planktonic form, which is not representative of the treatment of chronic wound infection as bacteria present in these infections are often in biofilm form. Therefore, it is instructive to test the anti-biofilm properties of the fibres.

**Fig. 2:**
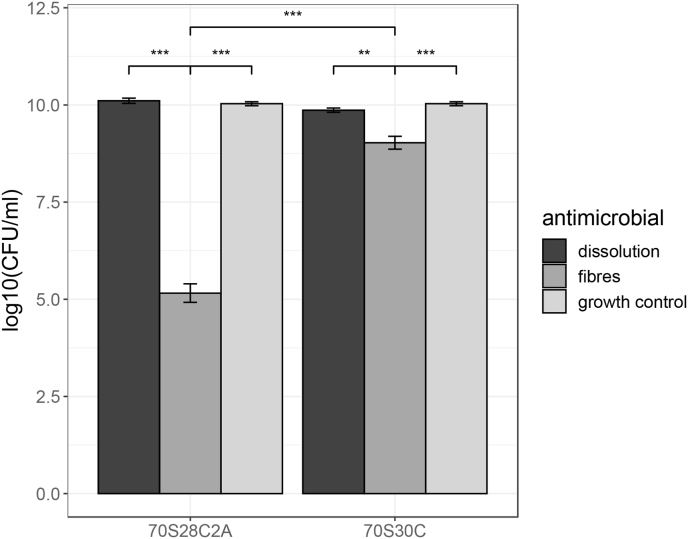
Comparing the effect of placing fibres on inoculated membranes and using dissolution products on biofilm inhibition. Biofilm growth under these conditions was compared to a growth control of an untreated 24hr biofilm. Error bars represent the average value ± 1 standard error of the mean. Statistical significance was calculated using a 1-way ANOVA, where b × p-value ≤ 0.05, 0.005, 0.0005 is represented by, *, **, *** respectively (where b = 8 using the Bonferroni correction to correct for multiple comparisons).

From Fig. 2, comparing the biofilm viability of a 24hr biofilm and biofilm formation when exposed to dissolution products, it can be seen that the dissolution products of both fibre compositions had minimal effect on inhibiting biofilm formation despite being able to strongly inhibit PAO1-N bacteria in planktonic form. This could be attributable to the fact that bacteria biofilms are more tolerant to antimicrobial pressure from dissolution products than their planktonic counterparts [[48], [49], [50]]. On the other hand, the direct application of fibres on the inoculated membrane resulted in a significant reduction of biofilm viability, especially for Ag-doped fibres, where the p-value was 3.31 × 10^−12^ for Ag-doped fibres and 2.51 × 10^−5^ for undoped fibres. For undoped fibres, the reduction of biofilm formation from direct contact of fibres rather than their dissolution products suggests that the fibres hold anti-biofilm properties through interaction between the fibres themselves and the biofilm causing physical disruption. This is corroborated by other research, where previous studies into the antimicrobial effects of BG found that direct contact between BG constructs and bacteria enhances antimicrobial effects against Gram-negative bacteria and that the glass formed “needle-like” structures which were able to damage the outer membrane of the bacteria [51,52]. Conversely, other studies have found that direct contact between BG constructs and bacteria does not have a significant effect on the antimicrobial activity, however the antimicrobial studies were only tested using Gram-positive bacterial species [53]. Therefore the Gram-type of the bacteria could affect whether BG fibres are able to physically damage the bacteria. Alternatively, it could be a consequence of local rises in pH and osmotic pressure in close proximity to the fibres instead of a more global rise from the dissolution products. For the Ag-doped fibre treatment on biofilm formation, there was over a 5log10 reduction in biofilm viability compared to no reduction when treating with dissolution products (Fig. 2). This could also be due to the interaction between the fibres and the biofilm itself and/or local rises in pH and osmotic pressure. Alternatively, the lack of anti-biofilm activity of the Ag-doped dissolution products could be due to the formation of silver chloride and/or silver sulphide. Therefore resulting in lower concentration of antimicrobial silver species, i.e. silver nanoparticles and Ag^+^ ions, and reducing the antimicrobial effects of the dissolution products.

### 3.3 Effect of silver chloride/sulphide formation on antimicrobial efficacy

One of the key issues with using Ag^+^ ions or Ag nanoparticles as a potential treatment is their ability to undergo multiple transformations in body fluid, which can depend on the pH, dissolved oxygen concentration, sunlight and temperature [42]. If the fluid contains high concentrations of chlorine and sulphur, which is often the case with simulated wound fluid, then silver chloride/sulphide can then form, which have minimal antimicrobial effect as they have low solubility [54,55]. Therefore, the possible formation of silver chloride/suphide when Ag-doped fibres are soaked in cell culture media was tested.

**Fig. 3:**
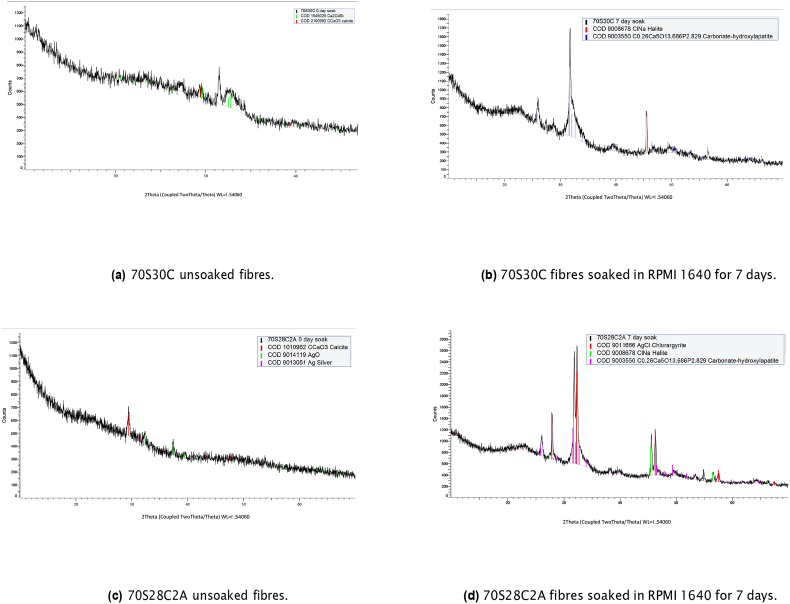
XRD of the 70S30C and 70S28C2A fibres either soaked in RPMI 1640 for 7 days before analysis or unsoaked. The presence of diffraction peaks indicates crystalline structures in the fibres. Fig. 3a and b demonstrate 70S30C fibres before and after soaking respectively and Fig. 3c and d demonstrate 70S28C2A fibres before and after soaking respectively.

Firstly, to determine whether silver chloride and/or silver sulphide forms whilst fibres are soaked in cell culture media XRD and TEM analysis was performed on fibres before and after soaking. From the XRD analysis (Fig. 3), it was observed that there were very few crystalline structures present in both fibre compositions before soaking. Calcite was present in both compositions, where characteristic peaks may be observed at 2 θ = 29.0° & 31.0° in Fig. 3a and c. Additionally, small amounts of calcium silicate were present in 70S30C fibres (2 θ = 29.4°, 32.2° & 32.5°). Further peaks in unsoaked 70S28C2A fibres represent either metallic silver, which has a characteristic peak of 2 θ = 37.4°, or silver oxide (characteristic peaks at 2 θ = 31.5°, 31.9° & 37.5°). After soaking in RPMI 1640 cell culture media for 7 days, both compositions contained strong presences of HCA formation on the fibres, where peaks were observed at 2 θ = 25.9°, 31.4° & 33.9° in Fig. 3b and d respectively and additional peaks occurred at 2 θ = 28.6° & 39.3° in soaked 70S30C fibres. HCA formation on bioactive glasses soaked in body fluids have been very well demonstrated [19]. This has been shown to occur via the supersaturation of fluid with respect to HCA formation due to the release of calcium ions within BG fibres as well as the uptake of both calcium and phosphorous from the cell culture media.

Additionally, sodium chloride was present in both fibre compositions after soaking, where visible peaks occur at 2 θ = 27.1°, 31.4°, 45.1° & 56.0°. This could be due to BG fibres retaining sodium chloride from the cell culture media because of incomplete washing. Interestingly, soaked 70S28C2A fibres showed no crystalline diffraction for metallic silver, instead, silver chloride was observed at 2 θ = 27.5°, 31.8°, 45.6°, 54.1° & 56.7° (Fig. 3d). This is due to Ag^+^ ions reacting with NaCl to form AgCl. There was no sign of silver sulphide formation.

**Fig. 4:**
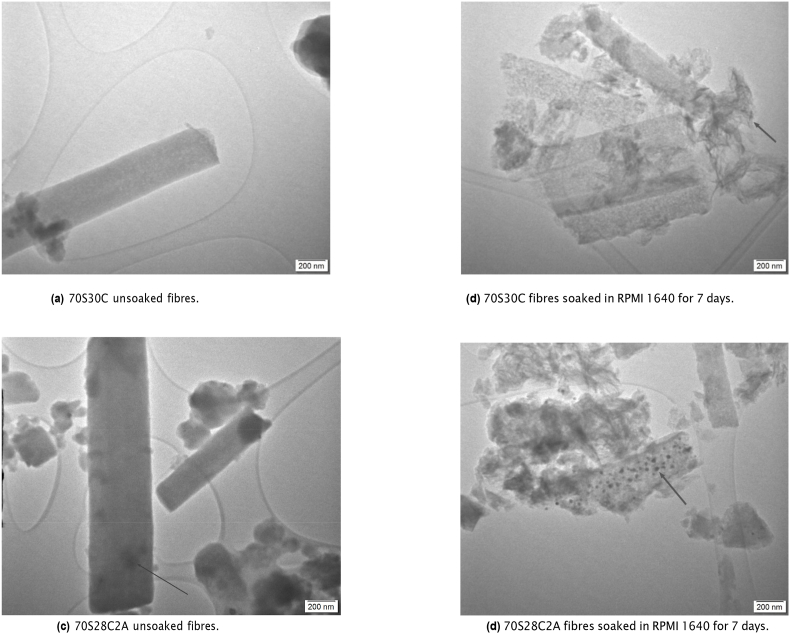
TEM images of the 70S30C and 70S28C2A fibres either soaked in RPMI 1640 for 7 days before imaging or unsoaked. Graphs show individual fibres and scale bars are 200 nm. Fig. 4a and b demonstrate 70S30C fibres before and after soaking respectively and Fig. 4c and d demonstrate 70S28C2A fibres before and after soaking respectively. Arrows on Fig. 4b and d indicate the locations from where SAED patterns were taken.

Fig. 4 illustrates TEM images of both fibre compositions before and after soaking. In Fig. 4a and 70S30C fibres appeared smooth and homogeneous. After soaking, fibres had a rough surface with protruding whiskers (see Fig. 4b). This was due to the formation of an HCA layer. Similarly, Fig. 4c and d shows that unsoaked 70S28C2A fibres appeared to be mostly smooth and homogeneous with some darker circles (arrow in Fig. 4c), which may be the presence of silver nanoparticles, whereas soaked fibres appeared to show the formation of needle-like HCA as well as more dark spots appearing within the fibres. This could once again be silver nanoparticles but could also be due to silver chloride (which was found to be present from XRD in Fig. 3d).

**Fig. 5:**
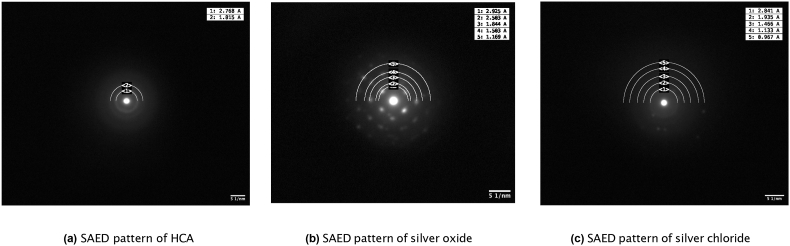
SAED patterns and measured d-spacing of fibres either soaked or not soaked in RPMI 1640 for 7 days. Fig. 5a is the corresponding diffraction pattern for soaked 70S30C fibres in Fig. 4b, where the diffraction pattern corresponds to HCA. Fig. 5b is the diffraction pattern for 70S28C2A fibres before soaking, where the d-spacing of rings corresponds to silver oxide. Finally Fig. 5c is the diffraction pattern for soaked 70S28C2A fibres seen in Fig. 4d, which identifies the crystal structure as silver chloride. Scale bars are of length 5 1/nm.

To identify crystalline structures within the TEM images SAED analysis was performed (Fig. 5). The needle/whisker shaped precipitates around the fibres (arrow in Fig. 4b) produced electron diffraction rings with d-spacing corresponding to HCA (Fig. 5a). Similar diffraction patterns were also observed in soaked 70S28C2A fibres (results were omitted as the diffraction pattern looked extremely similar to Fig. 5a). Additionally, observing the diffraction pattern in Fig. 5b, the dark particles found in images of unsoaked 70S28C2A (Fig. 4c) fibres had diffraction patterns corresponding to silver oxide. Finally, the particles in soaked 70S28C2A fibres in Fig. 4d (see arrow for the location from where the SAED pattern was taken) had diffraction patterns corresponding to silver chloride (see Fig. 5c). These results corroborate the results produced from the XRD analysis (Fig. 3). Although the presence of metallic silver in Ag-doped BG has been identified in other studies [28,56], here, electron diffraction patterns corresponding to metallic silver could not be observed. Due to a limitation of the SAED technique employed in this study; the central beam in the current setup could not be masked, this meant that some of the rings may have been masked by the high intensity central beam (see Supplementary Fig. S1 for additional SAED patterns of unsoaked 70S28C2A fibres, which demonstrate the difficulty in defining electron diffraction patterns due to the glare from the high intensity central beam).

From XRD and SAED analysis (Fig. 3, Fig. 5 respectively), it was demonstrated that when Ag-doped fibres are soaked in cell culture media, silver chloride forms but not silver sulphide. This may be due to the substantially higher concentrations of chlorine present in RPMI 1640 compared to sulphur, resulting in the domination of silver chloride formation. Moreover, other research has found that silver sulphidation is dominant over chlorination in anaerobic conditions [57]. This may also explain why there is no evidence of silver sulphide, as fibres were soaked in media in aerobic conditions.

**Fig. 6:**
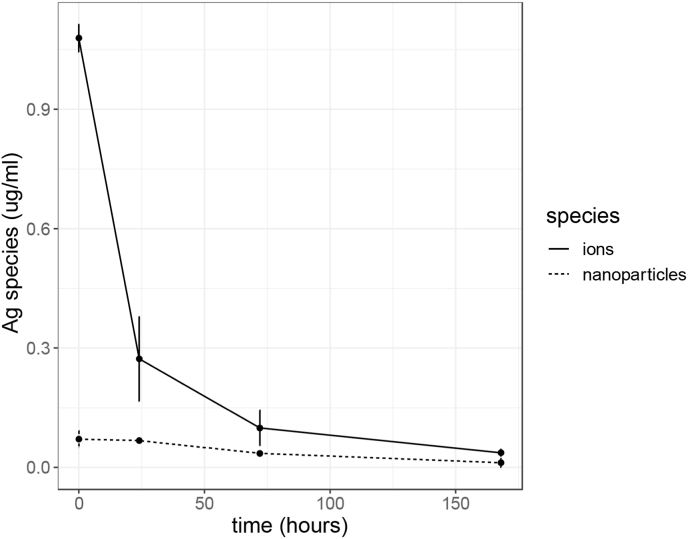
The change in silver ions and silver nanoparticle species concentration over time whilst dissolution products of 70S28C2A fibres are incubated at 37 °C. Error bars represent the average value ± 1 standard error of the mean.

As silver chloride forms, the concentrations of antimicrobial silver, namely silver nanoparticles and silver ions, are expected to reduce. To test this, the dissolution products of Ag-doped fibres were stored at 37 °C and were treated to separate and measure the different concentrations of antimicrobial silver over 7 days. Fig. 6 shows that both the silver ion concentration and the silver nanoparticle concentration decreased with time. This could be due to the formation of silver chloride which is shown from the XRD and TEM analysis (Fig. 3, Fig. 5c respectively). Moreover, there were higher concentrations of silver ions than silver nanoparticles.

**Fig. 7:**
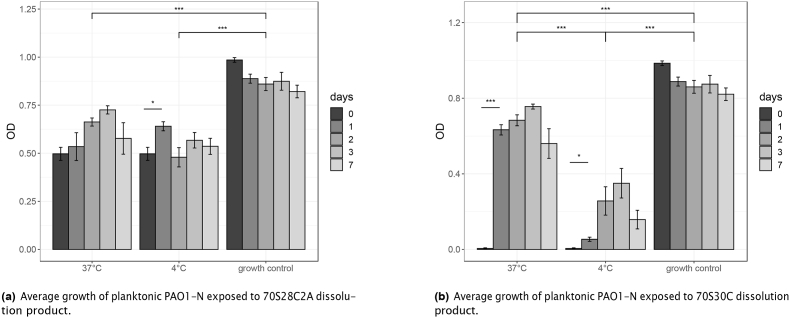
Growth of planktonic PAO1-N, when exposed to dissolution products of standard fibres (Fig. 7b) or fibres doped with silver (Fig. 7a). Graphs compare the effect of age on dissolution products' efficacy as well as the effect of the temperature of storage conditions. Error bars represent the average value ± 1 standard error of the mean. Statistical significance was calculated using a 1-way ANOVA, where b × p-value ≤ 0.05, 0.005, 0.0005 is represented by, *, **, *** respectively (where b = 11 using the Bonferroni correction to correct for multiple comparisons).

As the concentration of antimicrobial silver species reduced with time, this could potentially reduce the antimicrobial effects of the dissolution products. Fig. 7 illustrates the effect of both storage temperature and age of dissolution products on the growth of PA01-N measured in OD. A calibration curve was used to estimate the approximate CFU/ml (see Fig. S2 in supplementary material) where 1 OD = 8*.*789 × 10^8^ CFU/ml. Observing Fig. 7a, for Ag-doped fibres, dissolution products became less antimicrobial the longer they were stored before being applied as treatment against PA01-N, where there was a significant reduction in antimicrobial activity comparing dissolution products stored for 0 days compared to dissolution products stored for 1 day at both 37 °C and 4 °C, with respective p-values of 9.45 × 10^−11^ and 0.0041. However, after day 1, there was no significant decrease in antimicrobial activity due to variability in the data. Moreover, Ag-doped dissolution products stored at 4 °C were significantly more antimicrobial than products stored at 37 °C (p-value: 6.5 × 10^−11^). Reduction of the antimicrobial activity of the Ag-doped dissolution products with increased storage time and storage temperature may be due to a reduction in the concentration of antimicrobial silver species through chlorination. Chlorination of silver nanoparticles often involves two steps. First, the nanoparticles undergo oxidation and release Ag^+^ ions (equation (1)).

(1) 4Ag + O_2_ + 2H_2_O → 4Ag^+^ + 4OH^*−*^

Ag^+^ ions then go on to react with chlorine in the environment (equation (2)).

(2) Ag^+^ + NaCl → AgCl + Na^+^

Full chemical names are provided in Table 2.

**Table 2:** Chemical names corresponding to chemical formulas in equations (1), (2).

Chemical name	Chemical formula
Silver nanoparticles	Ag
Silver ions	Ag^+^
Oxygen	O_2_
Water	H_2_O
Hydroxide	OH^*−*^
Sodium chloride	NaCl
Silver chloride	AgCl
Sodium ions	Na^+^

Oxidation is the rate-limiting step and is more likely to occur with increasing dissolved oxygen in the fluid and increased temperature [42,[58], [59], [60]]. Therefore an increased temperature and consequently higher rate of oxidation results in a higher rate of silver chloride formation and may explain why dissolution products were less antimicrobial when stored at 37 °C compared to 4 °C. Furthermore, increased age of dissolution products means that there is more time for the redox reaction to occur, which may explain why there was a significant drop in antimicrobial activity between Ag-doped fibres stored for 0 days and stored for 1 day.

Moreover, observing Fig. 7b, for undoped fibres, it can be shown that the age of the dissolution products as well as the conditions they were stored in had very little effect on their antimicrobial activity against PAO1-N.

As ageing and storage temperature strongly affected only the antimicrobial efficacy of Ag-doped fibres it suggests that the reduction in antimicrobial efficacy is due to an effect on the silver population of the dissolution products. The reduction in antimicrobial silver is highly likely due to the formation of silver chloride, demonstrated from XRD and SAED analysis (Fig. 3, Fig. 5 respectively), which has little antimicrobial effect.

Therefore, this may partly explain why Ag-doped dissolution products had weaker anti-biofilm properties compared to the fibres themselves, as for silver ions, there was a competition for chlorination vs ions available for antimicrobial activity, where competition was in favour of chlorination with increased ageing time. A caveat of using dissolution products is that there was a period of time, whilst the fibres are soaked in media, that a proportion of released silver ions will chlorinate before the dissolution products are even applied to bacteria. Whilst for fibres that are directly applied to the inoculated membrane, ageing time was non-existent and thus correlates with activity.

However, silver chloride nanoparticles have often demonstrated strong antimicrobial effects against planktonic bacteria, mainly through the release of Ag^+^ ions, where their low solubility in water results in a slow and continuous Ag^+^ ion release rate [[61], [62], [63]], rather than a burst release demonstrated by other types of silver-based treatments, including silver nanoparticles, and the Ag-doped fibres in this study [28,56]. The main advantage of a continuous release is that, in a wound environment, concentrations of Ag^+^ ions could be low enough to avoid having cytotoxic effects and also reduce the chance of bacterial regrowth. However, in this study, the formation of silver chloride is detrimental to the antimicrobial effects of the dissolution products as silver chloride precipitates out of the solution due to its low solubility in water. Consequently, silver chloride formation results in dissolution products with lower concentration of silver in.

### 3.4 Importance of direct application of fibres on biofilms

Finally, the antimicrobial efficacy of fibres placed directly on inoculated membranes compared to fibres suspended just above the inoculated membranes in inserts was investigated. The inserts were placed in the wells so that the bottom of the insert was just touching the inoculated membrane, this was done so that fibres were kept in a similar proximity to the inoculated membrane compared to fibres placed directly on top with no insert. This allowed for a real-time release of silver and therefore minimising silver chloride formation without including physical disruption of the biofilm by fibres. The aim here was to see whether direct application of fibres, which could lead to physical disruption of the biofilm, plays an important part in inhibiting biofilm formation.

**Fig. 8:**
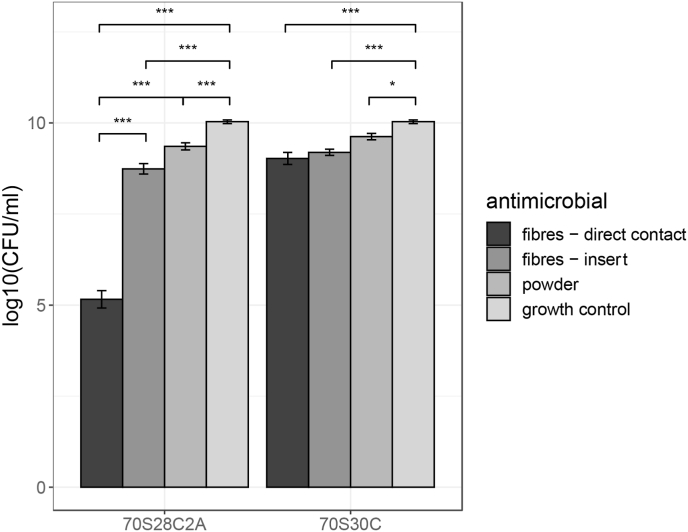
The effect of BG on PA01 biofilm inhibition. Both 70S30C and 70S28C2A compositions were tested either by placing fibres directly on top of the inoculated membrane, placing an insert suspended above the membrane, or using BG powder. Biofilm growth under these conditions was compared to a growth control of an untreated 24hr biofilm. Error bars represent the average value ± 1 standard error of the mean. Statistical significance was calculated using an 1-way ANOVA, where b × p-value ≤ 0.05, 0.005, 0.0005 is represented by, *, **, *** respectively (where b = 10 using the Bonferroni correction to correct for multiple comparisons).

From Fig. 8, it can be seen that fibres placed in inserts resulted in significantly lower biofilm viability compared to the growth control, where treatment using Ag-doped fibres and undoped fibres had p-values of 3.32 × 10^−12^ and 2.55 × 10^−5^ respectively. For the undoped fibres, there was no significant reduction in biofilm viability when fibres are placed directly on inoculated membranes compared to fibres placed in inserts above the biofilm.

**Fig. 9:**
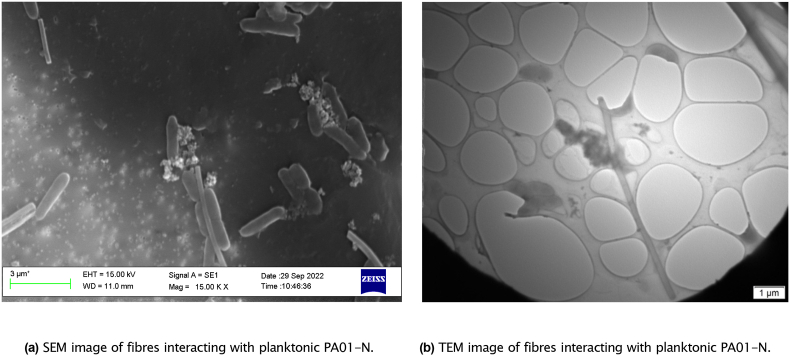
SEM and TEM images illustrating the interaction between BG fibres and planktonic PA01-N. From the images, it can be shown that fibres can physically disrupt the bacteria and cause leakage of intracellular components.

This suggests that in this case, local rises in pH (Fig. 1d) and osmotic pressure had stronger antimicrobial activity than any physical disruption caused by the fibres themselves. Therefore, whilst other literature has discovered that “needle-like” sharp debris from BG can damage the cellular structure of Gram-negative bacteria [51,52], this study has found that this effect is dominated by other means of antimicrobial activity. The effect of the undoped fibres directly on the bacteria is illustrated in Fig. 9. The SEM and TEM images show the rod-shaped structure of PA01-N. However, bacteria near or touching the fibres appear to have damaged structures with clear leakage of intracellular components. These images support that BG fibres can damage bacterial cells in close proximity, either through physical disruption, or local rises in pH and osmotic pressure.

On the other hand, for Ag-doped fibres, there was a significant reduction (p-value: 2.08 × 10^−9^) in biofilm viability when fibres were placed directly on top of the membrane compared to when they were placed in inserts. This indicates that, for Ag-doped fibres, direct contact between fibres and the inoculated membrane was crucial for inhibiting biofilm formation.

Moreover, there was a significant reduction in biofilm viability using BG powder treatment, where powdered BG was applied directly on top of the forming biofilm compared to the growth control, where the p-values for Ag-doped powder and undoped powder were 1.15 × 10^−5^ and 9.5 × 10^−4^ respectively. There was no difference in biofilm viability when comparing the effects of undoped BG powder and BG fibres. However, Ag-doped fibres reduced biofilm viability substantially more than Ag-doped BG powder (p-value: 6.42 × 10^−11^). Ag-doped BG in fibre form may have stronger anti-biofilm effects than BG in powder form as the 3D fibrous porous architecture of the fibres mean that they can wick up fluid, as discussed in Ref. [16]. As biofilms are predominately made of water [64], the fibres may be able to wick into the forming biofilm and release Ag directly into the biofilm, minimising diffusive effects. This could also explain why Ag-doped fibres placed in inserts could not inhibit biofilm formation as effectively, as the bottom of the insert acts as a barrier between the fibres and the forming biofilm. Therefore, the fibre structure for Ag-doped BG and contact with the forming biofilm is crucial for inhibiting biofilm formation.

## 4 Conclusion

Bioactive glass (BG) fibres offer a promising treatment for *P. aeruginosa* biofilms. BG fibres had stronger anti-biofilm effects than BG powder with the same composition and BG fibres doped with silver provided stronger anti-biofilm effects than undoped fibres. Importantly, the most effective form of treatment included the application of Ag-doped fibres directly on top of the inoculated surface. This minimised the amount of silver chloride formation as any released silver may target the forming biofilm before reacting with chlorine in the media, unlike with dissolution products, where released silver was exposed to chlorine in the media for a substantial amount of time before being applied to the biofilm. Furthermore, the direct application of Ag-doped fibres on inoculated surfaces allowed for a synergistic interaction between physical contact of the fibres on any forming biofilm alongside the release of antimicrobial silver, possibly as a consequence of the fibres facilitating release of silver directly within the biofilm. Future work will focus on investigating further why this synergistic interaction occurs.

Finally, the results of this study focused on inhibiting single-species biofilm formation. However, chronic wounds are likely to contain already established multi-species biofilms. Therefore, future work should include testing the antimicrobial efficacy of fibres on established multi-species biofilms.

## CRediT authorship contribution statement

**Sandeep Shirgill:** Conceptualization, Methodology, Software, Formal analysis, Investigation, Writing – original draft, Visualization. **Gowsihan Poologasundarampillai:** Conceptualization, Writing – original draft, Visualization, Supervision. **Sara Jabbari:** Conceptualization, Writing – original draft, Visualization, Supervision. **John Ward:** Conceptualization, Writing – original draft, Visualization, Supervision. **Sarah A. Kuehne:** Conceptualization, Writing – original draft, Visualization, Supervision, Project administration.

## Declaration of competing interest

The authors declare the following financial interests/personal relationships which may be considered as potential competing interests:Sandeep Shirgill reports financial support was provided by 10.13039/501100000268Biotechnology and Biological Sciences Research Council. Gowsihan Poologasundarampillai reports financial support was provided by 10.13039/501100000266Engineering and Physical Sciences Research Council.

## Figures and Tables

**Figure:**
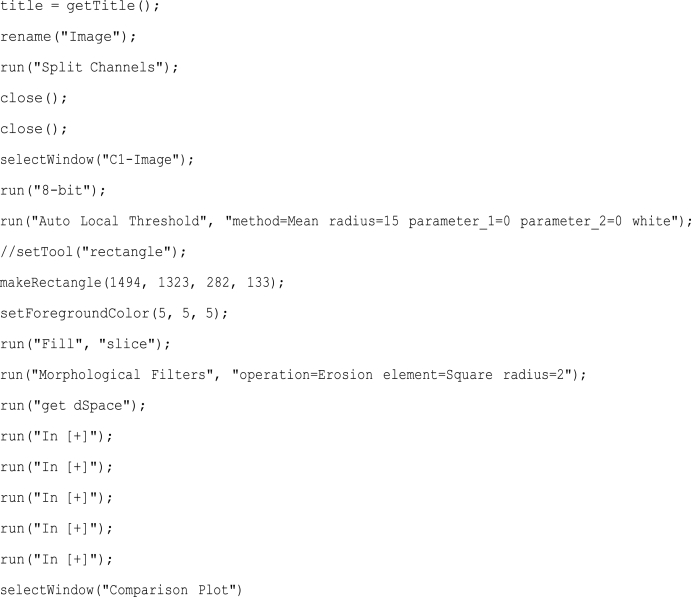


**Fig. S1a:**
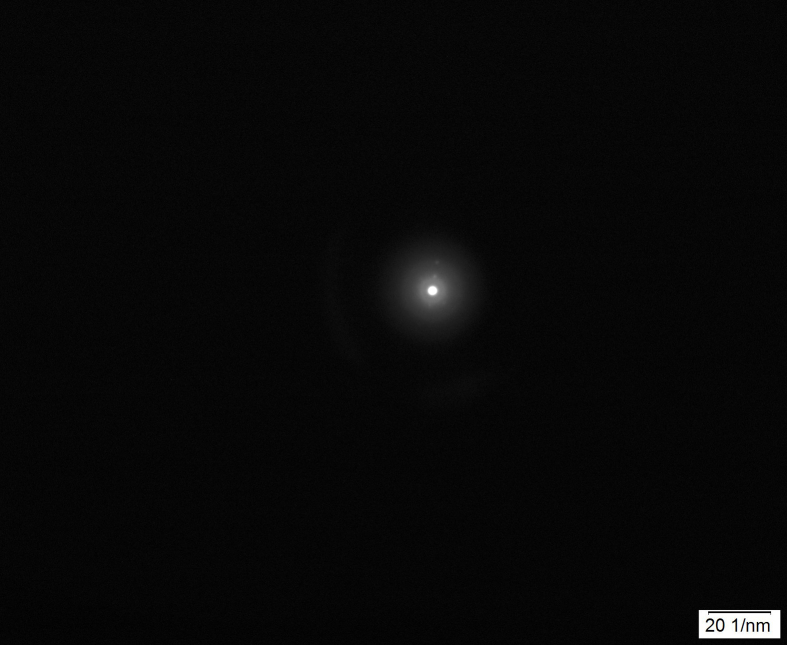
SAED patterns for unsoaked 70S28C2A fibres taken at different areas demonstrating the presence of diffused rings near the main transmitted beam.

**Fig. S1b:**
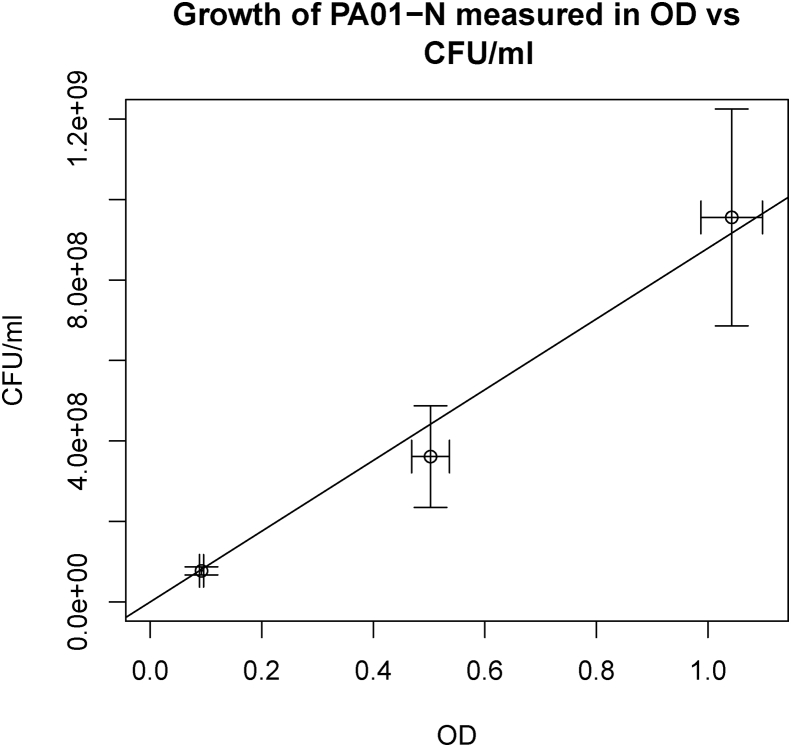
A calibration curve to determine the relationship between PA01-N growth measure in OD_600_ and in CFU/ml. From the graph, a line of best fit was used to determine that 1 OD = 8.789 x 10^8^CFU/ml with an R^2^ value of 0.9923.

## Data Availability

Data will be made available on request.

## References

[1] Kerr Marion (2011). Inpatient care for people with diabetes: the economic case for change. Tech. rep. London: NHS Diabetes.

[2] Julian F. Guest (2015). Health economic burden that wounds impose on the National Health Service in the UK”. In: BMJ Open.

[3] James Garth A. (2008). Biofilms in chronic wounds. In: Wound Repair Regen.

[4] Bowler P.G., Bi Duerden, Armstrong David G. (2001). Wound microbiology and associated approaches to wound management. In: Clin Microbiol Rev.

[5] Percival Steven L. (2012). A review of the scientific evidence for biofilms in wounds. In: Wound Repair Regen.

[6] Metcalf Daniel G., Bowler Philip G. (2013). Biofilm delays wound healing: a review of the evidence. In: Burns & Trauma.

[7] Wolcott R.D., Rhoads D.D. (2008). A study of biofilm-based wound management in subjects with critical limb ischaemia. In: J Wound Care.

[8] Stewart Philip S. (2002). Mechanisms of antibiotic resistance in bacterial biofilms. In: International journal of medical microbiology.

[9] Jones Julian R. (2013). Review of bioactive glass: from Hench to hybrids. In: Acta Biomater.

[10] Jones Julian R. (2016). Bioglass and bioactive glasses and their impact on healthcare. In: Int J Appl Glass Sci.

[11] Hench Larry L. (2006). The story of Bioglass. In: J Mater Sci Mater Med.

[12] Mao Cong, Cai Lin, Chen Xiaofeng (2014). Enhanced healing of full-thickness diabetic wounds using bioactive glass and Yunnan baiyao ointments. In: J Wuhan Univ Technol -Materials Sci Ed.

[13] Cai Lin (2012). Healing effect of bioactive glass ointment on full-thickness skin wounds. In: Biomed Mater.

[14] Gao Wendong (2017). A highly bioactive bone extracellular matrix-biomimetic nanofibrous system with rapid angiogenesis promotes diabetic wound healing. Int J Mater Chem B.

[15] Zhao Shichang (2015). Wound dressings composed of copper-doped borate bioactive glass microfibers stimulate angiogenesis and heal full-thickness skin defects in a rodent model. In: Biomaterials.

[16] Naseri Shiva, Lepry William C., Nazhat Showan N. (2017). Bioactive glasses in wound healing: hope or hype?. Int J Mater Chem B.

[17] Norris Elizabeth (2020). Electrospinning 3D bioactive glasses for wound healing. In: Biomed Mater.

[18] Gowsihan Poologasundarampillai and Akiko Obata. “Electrospun bioactive glass and organic-inorganic hybrid fibers for tissue regeneration and drug delivery”. In: Electrospun polymers and composites. Elsevier, pp. 77–110.

[19] Poologasundarampillai Gowsihan (2014). Cotton-wool-like bioactive glasses for bone regeneration. In: Acta Biomater.

[20] Hermans M.H.E., Cutting K. (2013). NPWT or HRT-dressing? Results of an expert panel and a Delphi panel analysis. In: J Wound Care.

[21] Whitney Kaitlyn E. (2017). Current perspectives on biological approaches for osteoarthritis. In: Ann N Y Acad Sci.

[22] Armstrong David G. (2022). A multi-centre, single-blinded randomised controlled clinical trial evaluating the effect of resorbable glass fibre matrix in the treatment of diabetic foot ulcers. In: Int Wound J.

[23] Jung Steven (2019). Anti-biofilm activity of two novel, borate based, bioactive glass wound dressings. In: Biomedical Glasses.

[24] Drago Lorenzo, Toscano Marco, Bottagisio Marta (2018). Recent evidence on bioactive glass antimicrobial and antibiofilm activity: a mini-review. In: Materials.

[25] Cora ca-Huber Débora C. (2014). Efficacy of antibacterial bioactive glass S53P4 against S. aureus biofilms grown on titanium discs in vitro. In: J Orthop Res.

[26] Romanó C.L. (2014). A comparative study of the use of bioactive glass S53P4 and antibiotic-loaded calcium-based bone substitutes in the treatment of chronic osteomyelitis: a retrospective comparative study. In: The bone & joint journal.

[27] Hoppe Alexander, Güldal Nusret S., Boccaccini Aldo R. (2011). A review of the biological response to ionic dissolution products from bioactive glasses and glass-ceramics. In: Biomaterials.

[28] Goh Yi-Fan (2014). Bioactive glass: an in-vitro comparative study of doping with nanoscale copper and silver particles. In: Int J Appl Glass Sci.

[29] Kaya Seray, Cresswell Mark, Boccaccini Aldo R. (2018). Mesoporous silica-based bioactive glasses for antibiotic- free antibacterial applications. In: Mater Sci Eng C.

[30] Deng-Guang Yu (2012). Polyacrylonitrile nanofibers coated with silver nanoparticles using a modified coaxial electrospinning process. In: Int J Nanomed.

[31] Maleki Homa, Mathur Sanjay, Klein Axel (2020). Antibacterial Ag containing core-shell polyvinyl alcohol-poly (lactic acid) nanofibers for biomedical applications. In: Polym Eng Sci.

[32] apa Agata L. (2019). Ga and Ce ion-doped phosphate glass fibres with antibacterial properties and their composite for wound healing applications. Int J Mater Chem B.

[33] Wilkinson Holly N. (2018). A novel silver bioactive glass elicits antimicrobial efficacy against Pseudomonas aeruginosa and Staphylococcus aureus in an ex vivo skin wound biofilm model. In: Front Microbiol.

[34] Paterson Thomas E. (2020). Multifunctional copper-containing mesoporous glass nanoparticles as antibacterial and proangiogenic agents for chronic wounds. In: Front Bioeng Biotechnol.

[35] Mekkawy Aml I. (2017). In vitro and in vivo evaluation of biologically synthesized silver nanoparticles for topical applications: effect of surface coating and loading into hydrogels. In: Int J Nanomed.

[36] Tiwari Vishvanath (2017). Effect of secondary metabolite of Actinidia deliciosa on the biofilm and extra-cellular matrix components of Acinetobacter baumannii. In: Microb Pathog.

[37] Harrison Joe J. (2004). Biofilm susceptibility to metal toxicity. In: Environ Microbiol.

[38] Chaloupka Karla, Malam Yogeshkumar, Seifalian Alexander M. (2010). Nanosilver as a new generation of nanoproduct in biomedical applications. In: Trends Biotechnol.

[39] Wilkinson L.J., White R.J., Chipman J.K. (2011). Silver and nanoparticles of silver in wound dressings: a review of efficacy and safety. In: J Wound Care.

[40] Xiaoxue Yin Iris (2020). The antibacterial mechanism of silver nanoparticles and its application in dentistry. In: Int J Nanomed.

[41] Liao Chengzhu, Li Yuchao, Chin Tjong Sie (2019). Bactericidal and cytotoxic properties of silver nanoparticles. In: Int J Mol Sci.

[42] Zhang Weicheng, Xiao Bangding, Fang Tao (2018). Chemical transformation of silver nanoparticles in aquatic environments: mechanism, morphology and toxicity. In: Chemosphere.

[43] Keith F Cutting (2003). Wound exudate: composition and functions. In: Br J Community Nurs.

[44] Mathew Joscilin, Sankar Parvathy, Varacallo Matthew (2021). StatPearls.

[45] Grazulis Saulius (2009). Crystallography Open Database–an open-access collection of crystal structures. In: J Appl Crystallogr.

[46] Jain Anubhav (2013). Commentary: the Materials Project: a materials genome approach to accelerating materials innovation. In: Apl Mater.

[47] Miles Ashley A., Misra S.S., Irwin J.O. (1938). The estimation of the bactericidal power of the blood. In: Epidemiol Infect.

[48] Monzón Marta (2002). Biofilm testing of Staphylococcus epidermidis clinical isolates: low performance of vancomycin in relation to other antibiotics. In: Diagn Microbiol Infect Dis.

[49] Helaine Sophie, Kugelberg Elisabeth (2014). Bacterial persisters: formation, eradication, and experimental systems. In: Trends Microbiol.

[50] Ayrapetyan Mesrop, Williams Tiffany C., Oliver James D. (2015). Bridging the gap between viable but non-culturable and antibiotic persistent bacteria. In: Trends Microbiol.

[51] Begum Saima (2016). The influence of pH and fluid dynamics on the antibacterial efficacy of 45S5 Bioglass. In: Biomed Mater.

[52] Hu Sheng (2009). Study on antibacterial effect of 45S5 Bioglass. In: J Mater Sci Mater Med.

[53] Allan I., Newman H., Wilson M. (2001). Antibacterial activity of particulate Bioglass® against supra-and sub-gingival bacteria. In: Biomaterials.

[54] Wright J Barry, Lam Kan, Burrell Robert E. (1998). Wound management in an era of increasing bacterial antibiotic resistance: a role for topical silver treatment. In: Am J Infect Control.

[55] Dunn Ken, Edwards-Jones Val (2004). The role of Acticoat™ with nanocrystalline silver in the management of burns. In: Burns.

[56] Ju Qun (2022). Silver-doped calcium silicate sol-gel glasses with a cotton-wool-like structure for wound healing. In: Biomaterials Advances.

[57] Jin Jiasheng (2023). Altering sliver nanoparticles-induced inhibition to bacterial denitrification via visible light by regulating silver transformation and adaptive mechanism under anaerobic conditions. In: Chem Eng J.

[58] Xiu Zong-Ming, Ma Jie, Alvarez Pedro JJ. (2011). Differential effect of common ligands and molecular oxygen on antimicrobial activity of silver nanoparticles versus silver ions. In: Environ Sci Technol.

[59] Liu Jingyu, Pennell Kelly G., Hurt Robert H. (2011). Kinetics and mechanisms of nanosilver oxysulfidation. In: Environ Sci Technol.

[60] Liu Jingyu, Hurt Robert H. (2010). Ion release kinetics and particle persistence in aqueous nano-silver colloids. In: Environ Sci Technol.

[61] Kang Yun Ok, Lee Taek Seung, Park Won Ho (2014). Green synthesis and antimicrobial activity of silver chloride nanoparticles stabilized with chitosan oligomer. In: J Mater Sci Mater Med.

[62] Min Seung-Hyun (2010). Development of white antibacterial pigment based on silver chloride nanoparticles and mesoporous silica and its polymer composite. In: Microporous Mesoporous Mater.

[63] Li Xianliang (2013). Silver chloride loaded hollow mesoporous aluminosilica spheres and their application in antibacterial coatings. In: Mater Lett.

[64] Quan Kecheng (2022). Water in bacterial biofilms: pores and channels, storage and transport functions. In: Crit Rev Microbiol.

