# Supplementary figures and images for: Silver-doped bioactive glass fibres as a potential treatment for wound-associated bacterial biofilms
Source: Biofilm. 2023 May 10;5:100115. doi: 10.1016/j.bioflm.2023.100115 (PMC10209705; doi:10.1016/j.bioflm.2023.100115)

# Growth of PA01–N measured in OD vs CFU/ml

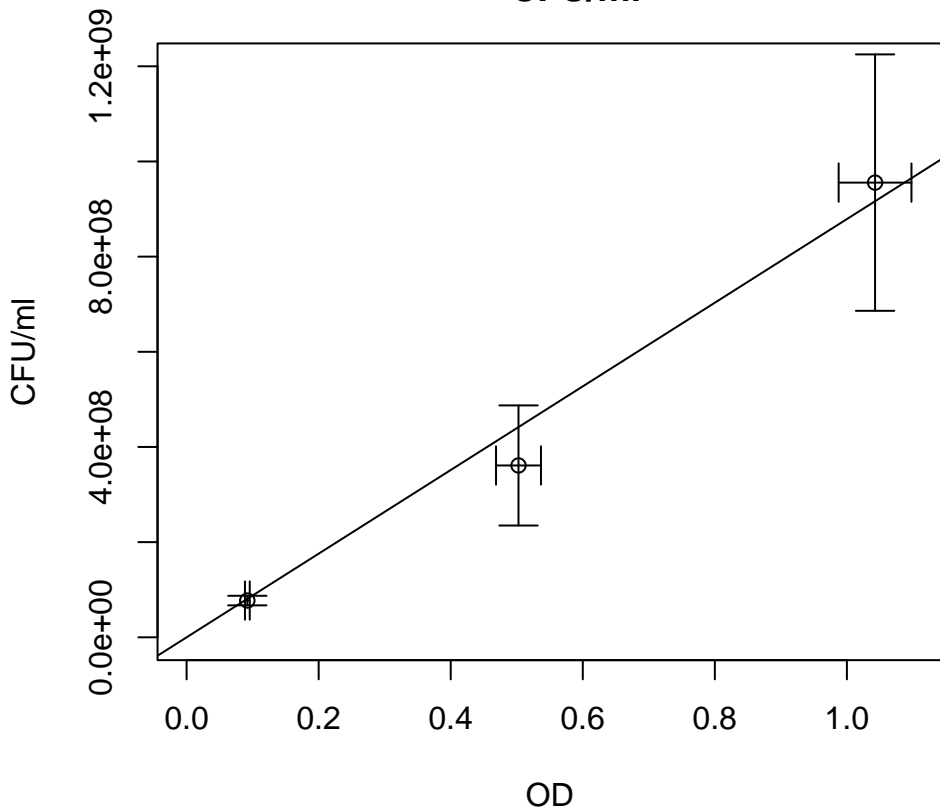

Supplement: Fig. S2 [file mmc1.pdf]
